# Single institution validation of a modified graded prognostic assessment of patients with breast cancer brain metastases

**DOI:** 10.2217/cns-2017-0023

**Published:** 2018-02-02

**Authors:** Cheng-Hung Tai, Cheng-Chia Wu, Mark E Hwang, Anurag Saraf, Christopher Grubb, Ashish Jani, Matthew E Lapa, Jacquelyn I S Andrews, Steven R Isaacson, Adam M Sonabend, Sameer A Sheth, Guy M McKhann, Michael B Sisti, Jeffrey N Bruce, Simon K Cheng, Eileen P Connolly, Tony JC Wang

**Affiliations:** 1 Department of Radiation Oncology, Columbia University Medical Center, New York, NY 10032, USA; 2 Department of Neurological Surgery, Columbia University Medical Center, New York, NY 10032, USA; 3 Herbert Irving Comprehensive Cancer Center, Columbia University Medical Center, New York, NY 10032, USA

**Keywords:** brain tumor, metastasis, radiation therapy

## Abstract

**Aim:**

The number of breast cancer brain metastases is a prognostic clinical variable in the modified graded prognostic assessment (GPA) Index for breast cancer.

**Patients & methods:**

We retrospectively gathered data from 127 breast cancer patients who underwent radiation therapy for brain metastasis. Patients were stratified by both breast GPA and modified breast GPA scores, and survival was determined using the Kaplan–Meier curves and Cox proportional hazards model.

**Results & Conclusion:**

The Kaplan–Meier curve for patients under the breast GPA classification were not significant, but were significant under the modified breast GPA classification. The inclusion of number of brain metastases into the modified breast GPA index improved prognosis, thus validating the use of the modified breast GPA in prognosticating patient outcome.

**Summary points**

- The breast cancer specific graded prognostic assessment (GPA) index for brain metastasis published by Sperduto *et al.* in 2012 found that the number of brain metastases (BM) was not a significant prognostic factor in a multi-institution study.
- A subsequent single institution analysis published by Subbiah *et al*. in 2015 found that the number of BM, in addition to Karnofsky Performance Score, age and breast cancer subtype, was prognostic of patient outcomes.
- Our single institutional analysis validates the modified breast GPA proposed by Subbiah *et al*. and affirms that the number of BM is a clinically significant variable that will guide appropriate radiotherapy treatment.
- Univariate analysis on each factor in breast and modified breast GPA found the number of BM, race, subtype and treatment modality to be significant prognostic factors.
- The patients accrued in this database were in a period where intracranial radiation outnumbered stereotactic radiosurgery, and with the evolution of radiotherapy treatment paradigms for patients with BM, predictive prognostic factor analyses may be potentially confounded.

Brain metastases (BM) carry significant risk and morbidity, signifying high tumor burden and advanced cancer stage [1,2]. Breast cancer is the second most common cause of BM. While 10–15% of patients with breast cancer develop BM in the course of their disease, autopsy studies suggest this proportion may be as high as 30% [3,4]. Yet, overall survival (OS) of subpopulations of breast cancer brain metastases (BCBM) patients has improved with advancements in systemic and radiation therapy, with improved prognostic indices informing personalized treatment options [5–8]. These treatment options include surgical resection, whole-brain radiation therapy (WBRT) and stereotactic radiosurgery (SRS).

Multiple indices comprising different diagnosis-specific prognostic factors have been compiled to predict OS of cancer patients with BM. BM risk factors in primary breast cancer include age, race, tumor burden, HER2/estrogen receptor (ER)/progesterone receptor (PR) status, *p53* gene expression and proliferation rate [9–13]. Variables thus commonly included in BCBM prognostic indices are age, Karnofsky Performance Score (KPS), tumor subtype, number of BM and presence of extracranial metastases (ECM).

Sperduto* et al.* developed the graded prognostic assessment (GPA) as a prognostic index for all cancer patients with BM in 2008 [14]. Compared with the Radiation Therapy Oncology Group's (RTOG) 1997 recursive partitioning analysis (RPA), the score-index for radiosurgery and the basic score for brain metastases (BSBM) using an RTOG BM database of 1960 patients, the GPA was as prognostic as the RPA and the least subjective of the four indices [15,16]. Factors found to be significant in the GPA were age, KPS, ECM and number of BM. The GPA was validated and subsequently modified as a disease-specific prognostic index for a number of primary cancers, including small and non-small-cell lung cancers, breast cancer, renal cell carcinoma and melanoma [17]. The breast GPA included two breast cancer-specific parameters: ER and PR hormone receptor (HR) status and EGFR2 (HER2) status [18]. Interestingly, number of BM was found not to be a significant prognostic factor in a multi-institutional review of 400 BCBM patients and was excluded from the breast GPA [17]. However, Subbiah* et al.* recently re-evaluated both the number of BM and the breast GPA as prognostic metrics in BCBM in a large single institution patient study and found that both independently predicted for OS. The proposed modified breast-GPA (henceforth the modified breast GPA) incorporated number of BM as the fourth clinical parameter in addition to age, KPS and cancer subtype [19].

As number of BM is a clinically important variable that guides treatment, and significant heterogeneity of size, geographic distribution and clinical practice exists in the patient cohorts in whom the breast GPA and modified breast GPA indices were evaluated, we sought to validate the modified breast GPA in our separate, single institution BCBM cohort.

## Patients & methods

Patients with metastatic brain tumors were retrospectively reviewed in our institutional review board (IRB) approved databases. From 1997 to 2015, a total of 1254 patients received wither Gamma Knife stereotactic radiosurgery or WBRT. Patients included in this study during primary initial staging did not present with BM, and had breast cancer with known molecular subtype proven through biopsy. MRI was the imaging modality used to confirm and evaluate the number of BM. All patients in the study were evaluated with brain MRI. Among the patients that were excluded include those that failed to complete their WBRT treatment course, and those that were simply lost to follow-up despite having completed their respective radiation treatment course. A total of 127 patients were identified that fit our criteria (Table 1).

**Table 1.** **Demographics.**

**Variables**	**All patients (n = 127), n**	**%**
Age at diagnosis:		

– ≤50	72	56.7

– >50	55	43.3

KPS:		

– ≤50	12	9.4

– 60	16	12.6

– 70–80	75	59

– 90–100	24	18.9

Race/ethnicity:		

– White	52	40.9

– Black	20	15.7

– Hispanic	30	23.6

– Other	25	19.7

BM:		

– 1–3	73	57.5

– >3	54	42.5

Subtype:		

– Receptor+/HER2+	33	26

– Receptor-/HER2+	15	11.8

– Receptor+/HER2-	57	44.9

– TN	22	17.3

Treatment:		

– WBRT	52	40.9

– SRS and WBRT	13	10.2

– Surgery and WBRT	13	10.2

– Surgery, SRS and WBRT	24	18.9

– SRS	25	19.7

Grade:		

– 1	10	7.9

– 2	67	52.8

– 3	19	15

– Unknown	31	24.4

Primary controlled:		

– Yes	66	52

– No	51	40.2

– Unknown	10	7.9

Chemotherapy:		

– Yes	99	78

– No	26	20.5

– Unknown	2	1.6

BM: Brain metastasis; KPS: Karnofsky Performance Score; SRS: Stereotactic radiosurgery; TN: Triple negative; WBRT: Whole-brain radiation therapy.

Breast GPA and modified breast GPA indices were calculated for each of 127 BCBM patients (Tables 2 & 3). Scoring in the Sperduto* et al.* breast GPA comprised KPS ≤50 (0 points), 60 (0.5 points), 70–80 (1.0 points) and 90–100 (1.5 points); triple-negative breast cancer (TNBC) subtype (0 points), HR+/HER2- (1 point), HR-/HER2+ (1.5 points), HR+/HER2+ (2 points); and age ≥60 years (0 points), <60 years (0.5 points).

**Table 2.** **Sperduto breast graded prognostic assessment score.**

**Variables**	**0–1 (n = 8)**		**1.5–2 (n = 36)**		**2.5–3 (n = 57)**		**3.5–4 (n = 26)**		**Pearson's χ^2^ p-value**

	**n**	**%**	**n**	**%**	**n**	**%**	**n**	**%**	
Age at diagnosis:									0.192

– ≤50	3	37.5	16	44.4	33	57.9	20	76.9	

– >50	5	62.5	20	55.5	24	42.1	6	23.1	

KPS:									< 0.001

– ≤50	2	25	9	25	1	1.8	0	0	

– 60	5	62.5	8	22.2	3	5.3	0	0	

– 70–80	1	12.5	15	41.7	43	75.4	16	61.5	

– 90–100	0	0	4	11.1	10	17.5	10	38.5	

Race/ethnicity:									0.31

– White	2	25	14	38.9	30	52.6	6	23.1	

– Black	3	37.5	7	19.4	6	10.5	4	15.4	

– Hispanic	2	25	10	27.8	10	17.5	8	30.8	

– Other	1	12.5	5	13.9	11	19.3	8	30.8	

BM:									0.435

– 1–3	4	50	21	58.3	30	52.6	18	69.2	

– >3	4	50	15	41.7	27	47.4	8	30.8	

Subtype:									< 0.001

– Receptor+/HER2+	0	0	0	0	7	12.3	26	100	

– Receptor-/HER2+	0	0	4	11.1	11	19.3	0	0	

– Receptor+/HER2-	1	12.5	17	47.2	39	68.4	0	0	

– TN	7	87.5	15	41.7	0	0	0	0	

Treatment:									0.429

– WBRT	6	75	19	52.8	21	36.8	6	23.1	

– SRS and WBRT	0	0	2	5.6	7	12.3	4	15.4	

– Surgery and WBRT	0	0	6	16.7	6	10.5	1	3.8	

– Surgery, SRS and WBRT	1	12.5	5	13.9	11	19.3	7	26.9	

– SRS	1	12.5	4	11.1	12	21.1	8	30.8	

Grade:									0.39

– 1	0	0	1	2.8	6	10.5	3	11.5	

– 2	5	62.5	25	69.4	27	47.4	10	38.5	

– 3	1	12.5	3	8.3	10	17.5	5	19.2	

– Unknown	2	25	7	19.4	14	24.6	8	30.8	

Primary controlled:									0.637

– Yes	5	62.5	18	50	30	52.6	13	50	

– No	3	37.5	17	47.2	20	35.1	11	42.3	

– Unknown	0	0	1	2.8	7	12.3	2	7.7	

Chemotherapy:									0.397

– Yes	6	75	26	72.2	44	77.2	23	88.5	

– No	2	25	9	25	13	22.8	2	7.7	

– Unknown	0	0	1	2.8	0	0	1	3.8	

BM: Brain metastasis; KPS: Karnofsky Performance Score; SRS: Stereotactic radiosurgery; TN: Triple negative; WBRT: Whole-brain radiation therapy.

**Table 3.** **Subbiah breast graded prognostic assessment score.**

**Variables**	**0–1 (n = 17)**		**1.5–2 (n = 50)**		**2.5–3 (n = 45)**		**3.5–4 (n = 15)**		**Pearson's χ^2^ p-value**

	**n**	**%**	**n**	**%**	**n**	**%**	**n**	**%**	
Age at diagnosis:									0.003

– ≤50	6	35.3	23	46	30	66.7	13	86.7	

– >50	11	64.7	27	54	15	33.3	2	13.3	

KPS:									< 0.001

– ≤50	7	41.2	5	10	0	0	0	0	

– 60	8	40.1	7	14	1	2.2	0	0	

– 70–80	2	11.8	34	68	33	73.3	6	40	

– 90–100	0	0	4	8	11	24.4	9	60	

Race/ethnicity:									0.492

– White	5	29.4	22	44	20	44.4	5	33.3	

– Black	4	23.5	7	14	8	17.8	1	6.7	

– Hispanic	6	35.3	12	24	8	17.8	4	26.7	

– Other	2	11.8	9	18	9	20	5	33.3	

BM:									0.007

– 1–3	5	29.4	25	50	28	62.2	15	100	

– >3	12	70.6	25	50	17	37.8	0	0	

Subtype:									< 0.001

– Receptor+/HER2+	0	0	1	2	17	37.8	15	100	

– Receptor-/HER2+	0	0	6	12	9	20	0	0	

– Receptor+/HER2-	9	52.9	31	62	17	37.8	0	0	

– TN	8	47.1	12	24	2	4.4	0	0	

Treatment:									0.043

– WBRT	13	76.5	20	40	17	37.8	2	13.3	

– SRS and WBRT	0	0	4	8	7	15.6	2	13.3	

– Surgery and WBRT	2	11.8	4	8	6	13.3	1	6.7	

– Surgery, SRS and WBRT	1	5.9	11	22	6	13.3	6	40	

– SRS	1	5.9	11	22	9	20	4	26.7	

Grade:									0.845

– 1	1	5.9	2	4	6	13.3	1	6.7	

– 2	11	64.7	28	56	23	51.1	5	33.3	

– 3	1	5.9	8	16	7	15.6	3	20	

– Unknown	4	23.5	12	24	9	20	6	40	

Primary controlled:									0.273

– Yes	10	58.8	22	44	28	62.2	6	40	

– No	7	41.2	25	50	11	24.4	8	53.3	

– Unknown	0	0	3	6	6	13.3	1	6.7	

Chemotherapy:									0.433

– Yes	12	70.6	38	76	37	82.2	12	80	

– No	5	29.4	11	22	8	17.8	2	13.3	

– Unknown	0	0	1	2	0	0	1	6.7	

BM: Brain metastasis; KPS: Karnofsky Performance Score; SRS: Stereotactic radiosurgery; TN: Triple negative; WBRT: Whole-brain radiation therapy.

Scoring in the Subbiah* et al.* modified breast GPA differed from the breast GPA in the following: subtype TNBC (0 points), HR+/HER2− (0.5 points), HR−/HER2+ (1.0 points); age >50 years (0 points), ≤50 years (0.5 points); and number of BM >3 (0 points), 1–3 (0.5 points). Patients were stratified into four subgroups in both the breast GPA and modified breast GPA with scores 0–1, 1.5–2, 2.5–3 and 3.5–4. Higher scores correlated with better prognosis.

Using the Kaplan–Meier estimator and Cox proportional hazards model, survival curves were calculated. To assess survival differences between each subgroup, log-rank tests were used. The OS time was measured from the last day of WBRT administration to the date of death or date of the last follow-up. For patients where the only first date of WBRT was known or recorded, the date in which treatment ended was approximated by adding ten business days after the initiation date. Furthermore, for 12 patients, only the month in which they initiated WBRT was known. As a result, the 15th of each month was used as an estimated treatment initiation date. Computations using the first, 15th and 30th day of each respective month as estimations were shown to not be significantly different. Patients lost to follow-up were censored for survival. For the 25 patients who received only SRS (Table 1), survival was determined as last SRS treatment date to date of death or date of last available follow-up. Prognostic factors were assessed through univariate and multivariate Cox proportional hazards regression and analyses. A p-value of less than 0.05 was defined as significant. All statistical analyses were performed using SPSS software.

## Results

**Figure 1.**
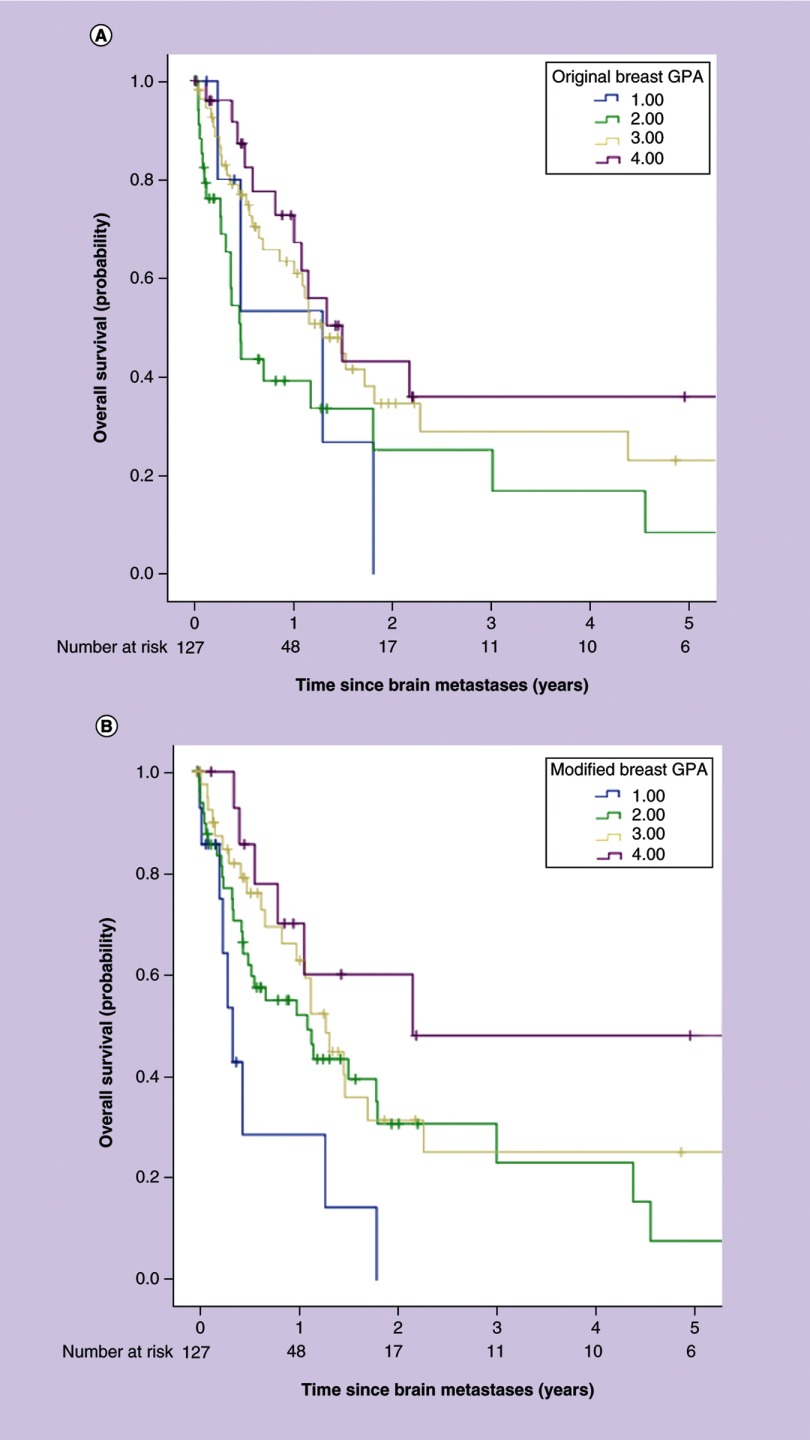
**Kaplan–Meier survical curves.** **(A)** Kaplan–Meier survival curves for original breast-GPA groups. Group 1: GPA: 0.0–1.0; MST: 1.29 years; (N = 8), group 2: GPA: 1.5–2.0; MST: 0.46 years; (N = 36), group 3: GPA: 2.5–3.0; MST: 1.30 years; (N = 57) and group 4: GPA: 3.5–4.0; MST: 1.49 years; (N = 26). Table 4 lists 1-, 2- and 3-year OS estimates and median OS in months by breast GPA group. **(B)** Kaplan–Meier survival curves for modified breast-GPA groups. Group 1: GPA: 0.0–1.0; MST: 0.362 years; (N = 17), group 2: GPA: 1.5–2.0; MST: 1.11 years; (N = 50), group 3: GPA: 2.5–3.0; MST: 1.30 years; (N = 45) and group 4: GPA: 3.5–4.0; MST: 2.17 years; (N = 15). Table 5 lists 1-, 2- and 3-year OS estimates and median OS in months by breast GPA group. GPA: Graded prognostic assessment; MST: Median survival time; OS: Overall survival.

Kaplan–Meier analysis with direct comparison of both statistical techniques is the statistical test that indicates that adding the number of BM improves the quality of the classification. Kaplan–Meier curves were stratified by breast GPA and modified breast GPA indices (Figure 1A & B). Median OS for all 127 patients was 418 days. Median OS for each adjusted modified breast GPA score of 0–1.0, 1.5–2.0, 2.5–3.0 and 3.5–4.0, was 132, 405, 473 and 791 days respectively (p = 0.013). Median OS for each adjusted breast GPA score of 0–1.0, 1.5–2.0, 2.5–3.0 and 3.5–4.0, was 470, 168, 473 and 543 days respectively (p = 0.081).

**Table 4.** **Original Breast GPA OS.**

**Original breast GPA**	**n (%)**	**OS, months (95% CI)**	**1-year OS, % (95% CI)**	**2-year OS, % (95% CI)**	**3-year OS, % (95% CI)**
0–1	8	15.5 (4.92–26.0)	53.3 (3.7–100)	0	0

1.5–2	36	5.5 (4.0–7.1)	39.2 (20.6–57.8)	25.2 (4.8–45.6)	25.2 (4.8–45.6)

2.5–3	57	15.6 (7.2–21.4)	63.3 (49.3–77.3)	34.6 (18.8–50.4)	28.8 (12.0–45.6)

3.5–4	26	17.9 (10.4–25.3)	72.7 (53.5–91.9)	43.2 (19.6–66.8)	36 (12.2–59.8)

GPA: Graded prognostic assessment; OS: Overall survival.

**Table 5.** **Modified Breast GPA OS.**

**Modified breast GPA**	**n (%)**	**OS, months (95% CI)**	**1-year OS, % (95% CI)**	**2-year OS, % (95% CI)**	**3-year OS, % (95% CI)**
0–1	17	4.3 (2.6–6.0)	28.6 (0–60.0)	0	0

1.5–2	50	13.3 (5.9–20.7)	55.0 (40.2–69.6)	30.7 (14.1–47.3)	30.7 (14.1–47.3)

2.5–3	45	15.6 (12.0–19.1)	66.2 (50.0–82.4)	31.4 (13.2–49.6)	25.1 (6.7–43.5)

3.5–4	15	26 (0–87.0)	70.1 (44.9–95.3)	60.1 (31.7–88.5)	48.1 (16.7–79.5)

GPA: Graded prognostic assessment; OS: Overall survival.

Univariate analysis was conducted on each factor included in both breast and modified breast GPA, in addition to race and treatment modality. Number of metastases, race, subtype and treatment modality (specifically WBRT) were found to be significant risk factors in both GPA classifications (Table 6). Receptor status subtype hazard ratios (HRs) were: HR+/HER2-, 2.486 (p = 0.018); HR-/HER2+, 1.305 (p = 0.383); and HR-/HER2-, 1.418 (p = 0.388). KPS was statistically significant (p = 0.004) at lower KPS scores with the following HRs: KPS: 70–80, 1.727 (p = 0.103); KPS: 60–69, 4.316 (p = 0.000); and KPS <60, 3.028 (p = 0.022). Finally, HR for number of metastases was 1.731 (p = 0.032) in patients with greater than three metastases versus patients with one-to-three metastases.

**Table 6.** **Univariate Cox proportional hazards model for overall survival (modified breast-graded prognostic assessment).**

**Variables**	**Hazard ratio**	**95% CI (lower)**	**95% CI (upper)**	**p-value**
Age at diagnosis:				

– ≤50	1			1

– >50	1.293	0.804	2.079	0.288

KPS:				

– 90–100	1			1

– 70–80	1.727	0.895	3.332	0.103

– 60	4.316	1.895	9.831	< 0.001

– ≤50	3.028	1.172	7.82	0.022

Race/ethnicity:				

– White	1			1

– Black	2.909	1.402	6.036	0.004

– Hispanic	1.39	0.719	2.685	0.328

– Other	1.045	0.571	1.912	0.886

BM:				

– 1–3	1			1

– >3	1.731	1.053	2.846	0.032

Subtype:				

– HR+/HER2+	1			1

– HR-/HER2+	2.486	1.168	5.291	0.018

– HR+/HER2-	1.305	0.717	2.374	0.383

– TNBC	1.418	0.642	3.134	0.388

Treatment:				

– SRS	1			1

– WBRT	2.628	1.371	5.035	0.004

– SRS and WBRT	0.865	0.344	2.173	0.758

– Surgery and WBRT	0.491	0.174	1.388	0.18

– Surgery, SRS and WBRT	0.808	0.376	1.739	0.586

BM: Brain metastasis; KPS: Karnofsky Performance Score; SRS: Stereotactic radiosurgery; TNBC: Triple-negative breast cancer; WBRT: Whole-brain radiation therapy.

## Discussion

Number of BM is a clinically significant variable that directs radiotherapy treatment. Its role as a prognostic factor in BCBM, however, has only been evaluated in two recent retrospective studies that disagree on statistical significance [19,20]. To date, our single institution experience of BCBM is the first to validate the modified breast GPA proposed by Subbiah *et al*. and show that number of BM contributes to the delineation of BCBM patients into four groups with significantly different prognoses.

The early diagnostic RPA and BSBM indices were not specific for breast cancer, and relied partly on subjective evaluation of patient functional status and control of primary tumor [21,22]. The initial GPA index (2008) was similarly not disease-specific but attempted to standardize prognostic evaluation of BM with more quantitative, rather than qualitative, parameters. Tumor burden was quantified by measuring both presence of ECM and number of CNS lesions. Presence of ECM notably comprised part of the prognostic evaluation of all three BM indices (RPA, BSBM and score index for radiosurgery) discussed in the original GPA publication [14].

Disease-specific GPA indices derived from the original GPA score were subsequently developed to evaluate BM prognosis in each of a number of primary malignancies. Number of BM was a preserved parameter in the GPA indices for non-small-cell lung cancer, small cell lung cancer, renal cell carcinoma and melanoma, as evaluated by Sperduto *et al.* [17]. The Barnholtz-Sloan *et al*. nomogram generated from an analysis of seven RTOG studies that combined predicting factors from the GPA and RPA indices also found number of brain lesions to be a significant prognostic factor in cancer BM [23].

Compared with the original 2008 GPA, the breast cancer-specific GPA (2012) included breast cancer receptor status and the concomitant removal of: number of BM; and ECM, from the index. Neither of the removed factors was found to be significant in the multi-institutional review of 400 BCBM patients. It is difficult to determine from the published analysis if these changes actually represented covariate redundancy following the addition of receptor status to the index.

Improved breast cancer systemic therapy was proposed to account for the apparent decline in significance of systemic disease burden as a BCBM prognostic factor in [20]. Indeed, the adoption of antihormonal therapy and HER2/neu targeted therapy into the standard of care, along with developments in conventional chemotherapy for patients with more advanced breast cancer, have led to increased distant metastasis-free survival and OS. However, this rationale is arguably less relevant to BM as the blood–brain barrier reduces the therapeutic impact of systemic therapy within the CNS.

Several small series have found the number of BM to be a significant prognostic factor in OS of BCBM patients in the past decade [24–30]. The clinical importance of number and location of BM is noteworthy as several published treatment algorithms consider these factors in the breast cancer treatment paradigm. For example, recommendations for HER2+ patients with 1–3 BM include SRS, whereas those with greater than three metastases include WBRT and/or palliative care [31,32]. Our single institution experience of 127 BCBM patients showed a strong correlation between patient outcomes and the modified breast GPA score. Most significantly, we identified a stratum of high performing patients in the modified breast GPA with OS >25 months that was well in excess of 18 months seen in with the best performing breast GPA stratum. In our patient cohort those who had the longest OS tended to be aged ≤50 (11 out of 16), have KPS >70 (13 out of 16) and <3 BM (14 out of 16).

Within the modified breast GPA index, significant factors on univariate analysis were KPS ≤50, number of BM, race, subtype and treatment modality, but not age. The latter have been repeatedly shown to be significant in previous, larger retrospective studies. A larger sample size is likely necessary to attain statistical significance for age. Multivariate analysis of our patient cohort was not possible for the same reasons.

Other limitations of this retrospective study include an institutional database comprising of only patients whose treatment included radiation therapy and not those who underwent systemic therapy alone. Further, patients were accrued to this database over a period in which intracranial radiation favored SRS over whole-brain radiation in a progressively larger proportion of patients. The effect of this evolution in the radiotherapy treatment paradigm for cancer BM patients on outcomes potentially confounds the impacts of prognostic with predictive factors, as the latter reflects treatment result rather than prognosis.

## Conclusion

The previous multi-institutional study by Sperduto *et al*. in 2012 found that the number of BM was not a significant prognostic factor in the breast cancer specific GPA. Subsequent analysis by Subbiah* et al.* found that in addition to KPS, breast cancer subtype and age, the number of BCBM was also a prognostic indicator of patient outcomes. Our data affirm that the inclusion of number of BCBM in the modified breast GPA index meaningfully contributes to prognostic stratification of BCBM patients. While our institutional experience validated the use of the modified breast GPA index, a multi-institutional prospective analysis should ultimately be conducted to confirm these results, and lead to new guidelines in treatment management.
